# Mesoglycan connects Syndecan‐4 and VEGFR2 through Annexin A1 and formyl peptide receptors to promote angiogenesis *in vitro*

**DOI:** 10.1111/febs.16043

**Published:** 2021-06-22

**Authors:** Emanuela Pessolano, Raffaella Belvedere, Nunzia Novizio, Amelia Filippelli, Mauro Perretti, James Whiteford, Antonello Petrella

**Affiliations:** ^1^ Department of Pharmacy University of Salerno Fisciano Italy; ^2^ The William Harvey Research Institute Barts and The London School of Medicine and Dentistry Queen Mary University of London UK; ^3^ Department of Medicine, Surgery, and Dentistry University of Salerno Baronissi Italy

**Keywords:** angiogenesis, annexin A1, mesoglycan, syndecan‐4, vascular endothelial growth factor

## Abstract

Mesoglycan is a mixture of glycosaminoglycans (GAG) with fibrinolytic effects and the potential to enhance skin wound repair. Here, we have used endothelial cells isolated from wild‐type (WT) and Syndecan‐4 null *(Sdc4‐/‐)* C57BL/6 mice to demonstrate that mesoglycan promotes cell motility and *in vitro* angiogenesis acting on the co‐receptor Syndecan‐4 (SDC4). This latter is known to participate in the formation and release of extracellular vesicles (EVs). We characterized EVs released by HUVECs and assessed their effect on angiogenesis. Particularly, we focused on Annexin A1 (ANXA1) containing EVs, since they may contribute to tube formation *via* interactions with Formyl peptide receptors (FPRs). In our model, the bond ANXA1‐FPRs stimulates the release of vascular endothelial growth factor (VEGF‐A) that interacts with vascular endothelial receptor‐2 (VEGFR2) and activates the pathway enhancing cell motility in an autocrine manner, as shown by wound healing/invasion assays, and the induction of endothelial to mesenchymal transition (EndMT). Thus, we have shown for the first time that mesoglycan exerts its pro‐angiogenic effects in the healing process triggering the activation of the three interconnected molecular axis: mesoglycan‐SDC4, EVs‐ANXA1‐FPRs, and VEGF‐A‐VEGFR2.

## Graphical abstract

The mesoglycan administered to endothelial cells interacts with Syndecan‐4 (SDC4) and this causes an increase in formation and secretion of vesicles containing the protein Annexin A1 (ANXA1). ANXA1, released through extracellular vesicles (EVs), interacts with Formyl peptide receptors (FPRs) in an autocrine way. This interaction promotes the release of Vascular endothelial growth factor (VEGF), which binds its receptor VEGFR2 enhancing the pro‐angiogenic activity in endothelial cells.

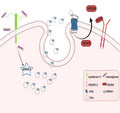

**Abbreviations**
ANXA1: annexin A1
EC: endothelial cell
ECM: extracellular matrix
EndMT: endothelial to mesenchymal transition
EVs: extracellular vesicles
FPRs: formyl peptide receptors
GAGs: glycosaminoglycans
HUVEC: human umbilical vein endothelial cells
MLEC: primary mouse lung endothelial cell
SDC4: syndecan‐4
*Sdc4-/-*
: syndecan-4 null mice
SEM: standard error of mean
siRNA: small interfering RNA
VEGF-A: vascular endothelial growth factor
VEGFR2: vascular endothelial growth factor receptor-2
WT: wild-type

## Introduction

Mesoglycan represents a potential therapy for augmenting the healing of skin lesions. This GAG mixture can promote angiogenesis *in vitro*, improving the generation of capillary‐like structures essential for wound repair process [1]. Angiogenesis is fundamental in many physiological and para‐physiological processes, and in the context of skin lesions, it plays a key role in providing the tissue with the necessary nutrients [2]. During this process, endothelial cells undergo the endothelial‐to‐mesenchymal transformation (EndMT). This is a mechanism in which cells are transformed from endothelial to mesenchymal phenotype as evidenced by loss of endothelial cell (EC) junctions, downregulation of endothelial markers and concomitant up‐regulation of mesenchymal markers leading to the acquisition of migratory properties favouring angiogenesis [3]. During wound healing, angiogenic capillaries infiltrate in the extracellular matrix (ECM). These cells arrange themselves into a microvascular network, produce granulation tissue [4] and lead to wound resolution resulting in healthy new skin. The formation of new vessels has a pivotal role in wound repair because the most common causes of the formation of chronic injuries are a poor perfusion of the wound, which leads to a diminished bioavailability of growth factors and receptors and a decreased proliferative potential of the cells at the injury site [5].

Vascular endothelial growth factors (VEGF‐A) promote several steps of wound healing such as collagen deposition, angiogenesis and epithelization [6]. There are five different isoforms of VEGF: VEGF‐A (VEGF_165_) VEGF‐B, VEGF‐C, VEGF‐D and PLGF. Among these isoforms, VEGF‐A165 is the most significant and it binds to both vascular endothelial growth factor receptors VEGFR‐1 and VEGFR‐2 [7]. The engagement VEGF‐A with VEGFR2 triggers numerous pro‐angiogenic pathways in ECs including the stimulation of vascular permeability, the loss of connections between endothelial cells and migration [8].

AnnexinA1 (ANXA1) and its mimetic N‐terminal peptide Ac2‐26 have caused considerable interest for its role in promoting angiogenesis [9, 10, 11]. Our research has highlighted that secreted ANXA1 contained within extracellular vesicles (EVs) stimulate the formation of capillary‐like structures *in vitro* [12]. Of relevance, the interaction between mesoglycan and Syndecan‐4 (SDC4) promotes a greater release of EVs containing ANXA1 in human keratinocytes [13, 14] and is potentially a paracrine way in which HUVEC tubulogenesis can be promoted [15].

Here, we explore, for the first time, the effects on endothelial cells of mesoglycan‐VEGF, investigating the association between two molecules that independently augment wound healing and are pro‐angiogenic. This study aims to understand the link between mesoglycan/SDC4 and VEGF‐A/VEGFR2 to establish connections between these two different pro‐angiogenic pathways.

## Results

### Mesoglycan and VEGF‐A in concert promotes angiogenesis *in vitro*

**Fig. 1:**
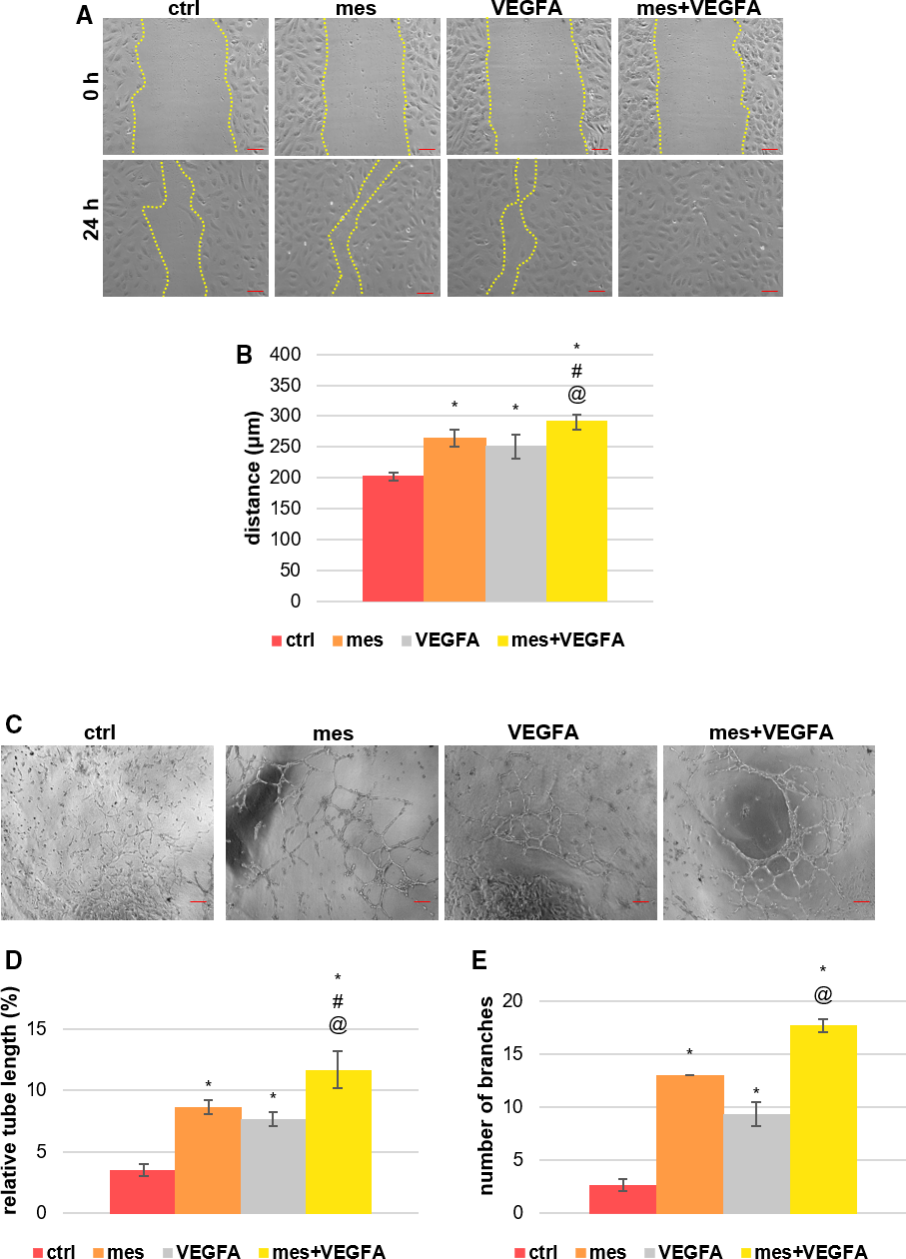
Evaluation of migration and tube formation of endothelial cells in response to VEGF‐A and mesoglycan (A) Representative images of scratch wound migration assays at the times indicated. Cells were treated with either mesoglycan (0.3 mg·mL^−1^), VEGF‐A (10 ng·mL^−1^), or a combination of the two. Scale bar = 150 μm. (B) The rate of cell migration was determined by measuring the wound closure by individual cells from the initial time (0 h) to the selected time‐points (24 h). Magnification 10×. (C) Representative images of tube formation by HUVEC cells seeded for 12 h on matrigel and EBM2 1 : 1 and in the presence or absence of mesoglycan (0.3 mg·mL^−1^), VEGF‐A (10 ng·mL^−1^), mesoglycan (0.3 mg·mL^−1^), and VEGF‐A (10 ng·mL^−1^). Scale bar = 100 μm. Analysis of (D) tube length and (E) number of branches calculated by ImageJ (Angiogenesis Analyzer tool) software. The data represent a mean of three independent experiments ± SEM. *P < 0.05 for all treatments vs. untreated cells. ^#^ *P* < 0.05 for all treatments vs. mesoglycan. ^@^ *P* < 0.05 for all treatments vs. VEGF‐A treatment.

Angiogenesis is the process that creates new blood vessels and plays a key role in wound repair [14]. Cell migration is an important angiogenic process and, we tested the effect of mesoglycan and VEGF‐A, a potent angiogenic factor, alone and in combination on the migratory properties of HUVECs using a scratch wound assay. As expected, treatment with VEGF‐A or mesoglycan leads to increased EC migration. Representative images of wound healing assay on HUVEC cells are reported in Fig. 1A, and surprisingly, effect on motility was increased further when both mesoglycan and VEGF‐A were used in combination (Fig. 1A,B). We next evaluated the effects of mesoglycan and VEGF‐A on micro‐capillary formation in the presence of matrigel. In common with the scratch wound assays, both VEGF‐A and mesoglycan treatment lead to the formation of significantly longer and more branched tubules as compared with the untreated control. These parameters were enhanced further when treated with VEGF‐A and mesoglycan in combination, as shown in Fig. 1C–E.

### The association between mesoglycan and VEGF‐A activates the VEGFR2 pathway in HUVEC cells

**Fig. 2:**
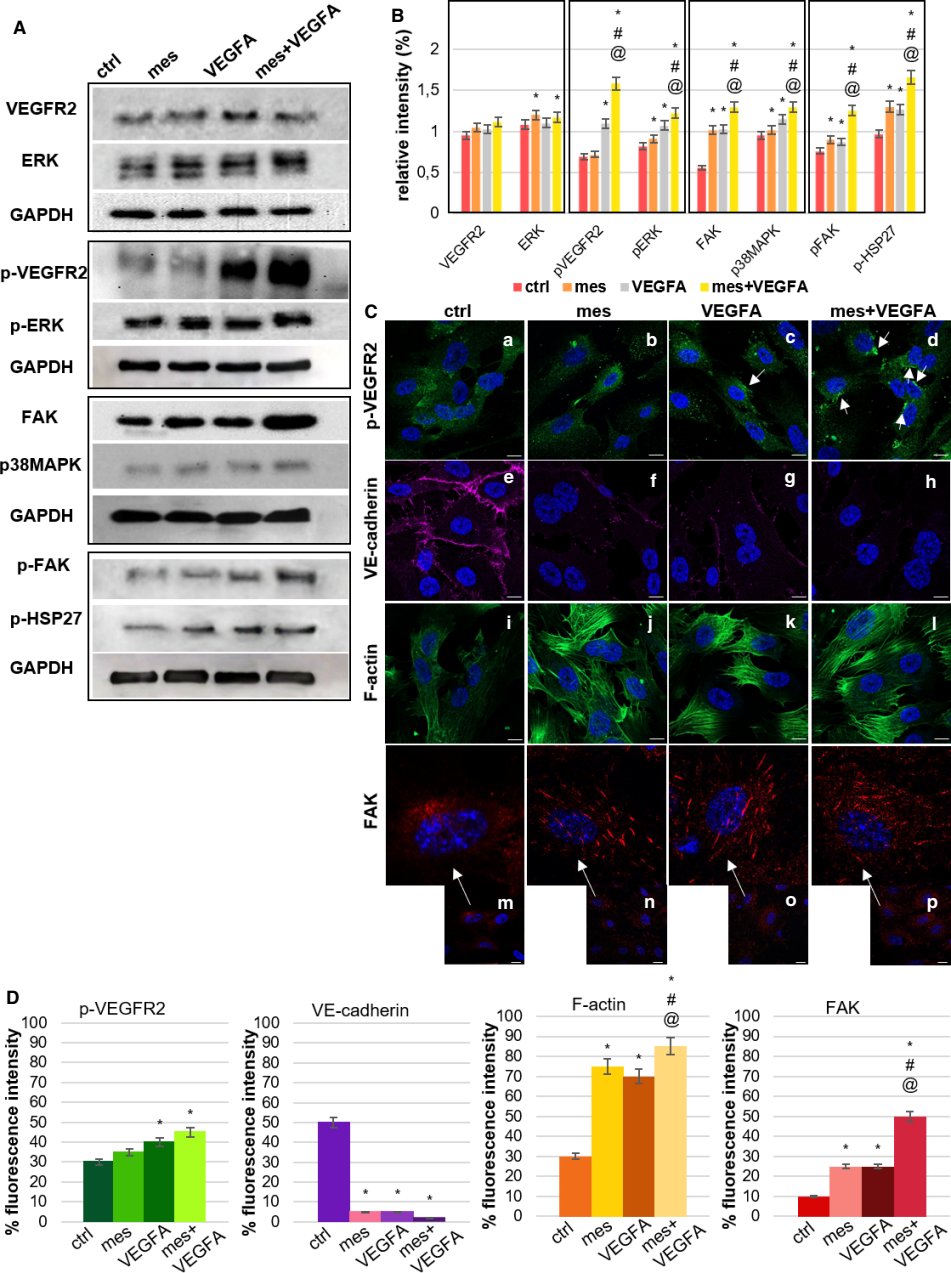
Analysis of VEGFR2 pathway in HUVEC cells (A) Western blot analysis and (B) quantification of total protein extracts from endothelial cells treated for 24 h with mesoglycan (0.3 mg·mL^−1^), VEGF‐A (10 ng·mL^−1^) or mesoglycan (0.3 mg·mL^−1^), and VEGF‐A (10 ng·mL^−1^) in combination. Cropped blots from full‐length gels are representative of *n* = 3 independent experiments with similar results using antibodies against VEGFR2, p‐VEGFR2, ERK, p‐ERK, p38MAPK, p‐HSP27, FAK, p‐FAK and normalized with GAPDH. The blots were exposed to Las4000 (GE Healthcare Life Sciences). The data represent a mean of three independent experiments ± SEM. *P < 0.05 for all treatments vs. untreated cells. ^#^ *P* < 0.05 for all treatments vs. mesoglycan. ^@^ *P* < 0.05 for all treatments vs. VEGF‐A treatment. (C) Immunofluorescence analysis of HUVEC cells in presence or not of mesoglycan (0.3 mg·mL^−1^), VEGF‐A (10 ng·mL^−1^) and mesoglycan (0.3 mg·mL^−1^) and VEGF‐A (10 ng·mL^−1^) co‐administrated. The cells were fixed and labelled with antibody against p‐VEGFR2 (panels a–d), VE‐cadherin (panels e–h), FAK (panels m–p, and relative 4× zoom) and with phalloidin (panels i–l). Nuclei were stained with Hoechst 33342 1 : 1000 for 30 min at room temperature (RT) in the dark. Magnification 63 × 1.4 NA. Scale bar = 50 μm. (D) Fluorescence intensity for p‐VEGFR2, VE‐cadherin, F‐actin and FAK signals on HUVEC cells using ImageJ software. The measurements are determined on ten field images from a single coverslip and randomly selected for three coverslips. *P < 0.05, vs. untreated control; ^#^ *P* < 0.05 for all treatments vs. mesoglycan. ^@^ *P* < 0.05 for all treatments vs. VEGF‐A treatment.

To explore the effect of mesoglycan and VEGF‐A in combination in stimulating angiogenesis, we explored the impact of these treatments on VEGFR2 signalling and effectors downstream of this receptor. Figure 2 showed that the treatment of HUVECs with VEGF‐A and mesoglycan alone or in combination had no discernible effects on the expression levels of VEGFR2. Engagement of VEGRF2 with VEGF‐A leads to the phosphorylation of a number of Tyr residues in its cytoplasmic domain. Phosphorylation of Tyr951 is intimately associated with EC migration [8]. Phosphorylation of this residue was comparable to controls when HUVECs were treated with mesoglycan alone; however, as expected VEGF‐A treatment resulted in an increase in phosphorylation of Tyr951 and this effect was even greater when mesoglycan and VEGF‐A were used in combination, which is suggestive of a role for mesoglycan in promoting the interaction between VEGF‐A and VEGFR2.

We next looked at the phosphorylation status of several downstream kinases of VEGFR2, which are known to be phosphorylated in response to VEGF‐A. These included, extracellular signal‐regulated kinases (ERK), p38 mitogen‐activated protein kinases (p38MAPK), heat shock protein 27 phosphorylation (p‐HSP27) and focal adhesion kinase (FAK). In all instances, phosphorylation was substantially more when mesoglycan and VEGF‐A were used in concert. As reported in the western blot in Fig. 2A and confirmed in the relative intensity analysis in Fig. 2B, no significant difference appeared in ERK expression between the treatments. In contrast, there was a positive regulation of p‐ERK in HUVEC treated with mesoglycan and VEGF‐A separately, but particularly when these two components were co‐administered. The same trend occurred for the expression levels of p38MAPK and p‐HSP27. p38MAPK‐HSP27 signalling downstream VEGF‐A‐VEGFR2 contributes to actin cytoskeleton reorganization and migration of endothelial cells to promote pro‐angiogenic effects [16]. FAK and its phosphorylated form (p‐FAK) are proteins downstream VEGFR2 pathway, involved focal adhesions and stress fibre development. The co‐administration of mesoglycan and VEGF promoted higher expression levels of FAK, and this was also reflected in an increase in p‐FAK. Interestingly, administration of VEGF‐A and mesoglycan alone could promote this increase in FAK levels (Fig. 2A,B).

Immunofluorescence analysis revealed that internalization of p‐VEGFR2 after 24 h of VEGF‐A treatment and this was enhanced by co‐administration with mesoglycan (Fig. 2C panels a–d). The quantification of the fluorescence intensity of p‐VEGFR2 shown in Fig. 2C (panels a–d) is reported in Fig. 2D. Loss of VE‐cadherin is associated with VEGF‐A promoted EndMT [17], and we observed a substantial loss of VE‐cadherin expression in HUVECS upon treatment with VEFA and mesoglycan (Fig. 2C panels e–h and Fig. 2D). The corresponded to an increase in the formation of stress fibres observed through the polymerization of F‐actin (Fig. 2C panels i–l) and FAK‐positive focal adhesions (Fig. 2C panels m–p). These data can be further appreciated in Fig. 2D. These results confirm that mesoglycan in concert with VEGF‐A leads to enhanced angiogenic responses and this is due to enhanced signalling through the VEGF‐A/VEGFR2 axis.

### Syndecan‐4 is required for mesoglycan‐VEGF‐A pro‐angiogenic responses *in vitro*

Our published study [14] describes how mesoglycan can stimulate syndecan‐4 pathway and promote the migration in human keratinocytes during wound repair process.

Recent evidence shows that SDC4 plays a key role in the regulation of cell migration. Analyses carried out on myoblasts show how, following the silencing of SDC4, the migration speed is strongly reduced [18]. Given the ubiquitous expression of SDC4, these results are potentially relevant to other cell types, such as in keratinocytes, as we have shown previously [14].

Moreover, mice null for SDC4 exhibit wound defects, particularly in the formation of granulation tissue in which angiogenesis is impaired and in wound closure [19]. Based on this, we speculated it may have a role in the synergistic effects of mesoglycan‐VEGF‐A on angiogenesis.

We therefore performed scratch wound migration assays on primary mouse lung endothelial cells from WT and *Sdc4‐/‐* animals. WT primary mouse lung endothelial cell (MLECs) had the same response profile we observed in HUVECs, in that co‐administration of VEGF‐A and mesoglycan significantly increased EC migration.

**Fig. 3:**
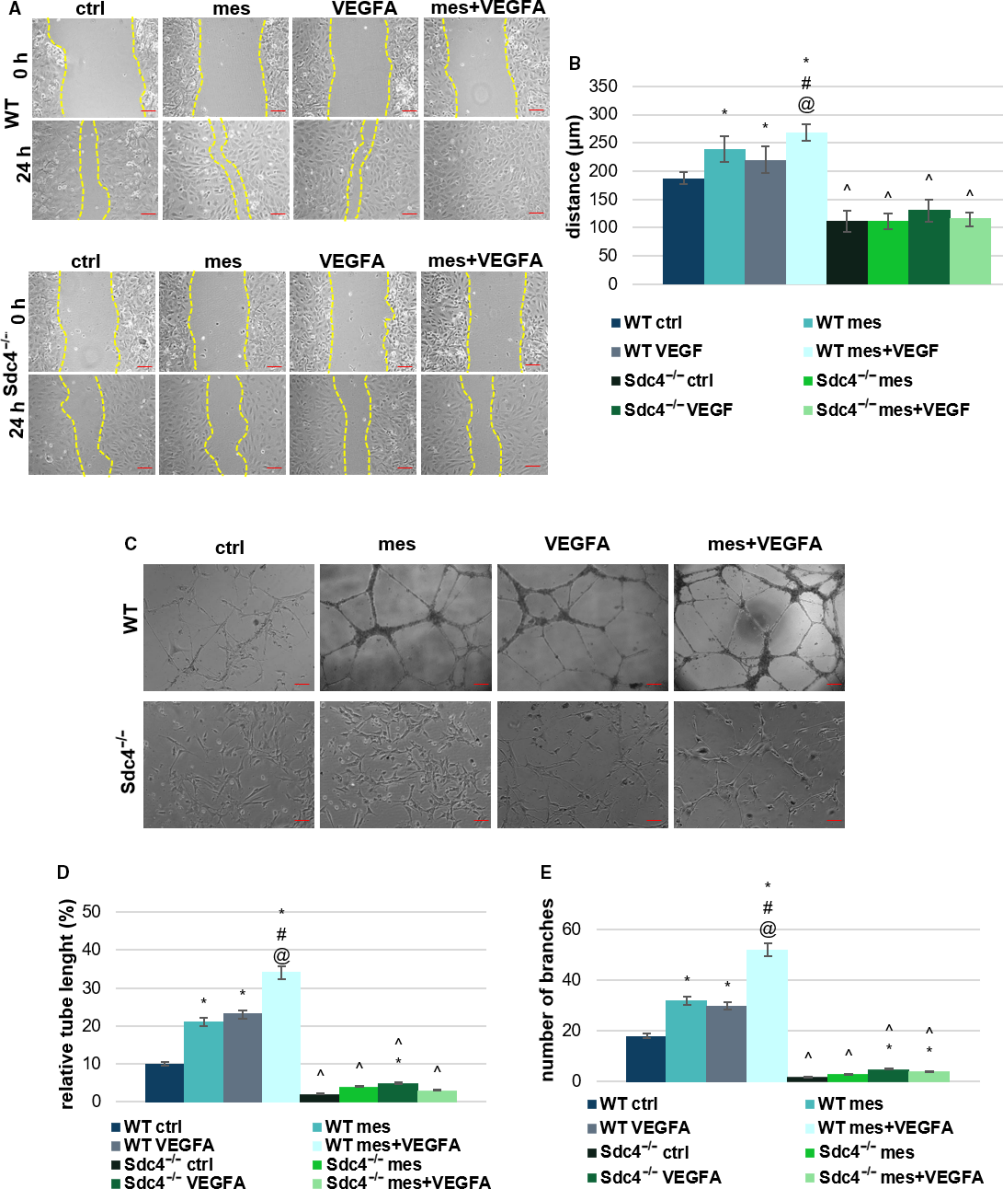
Evaluation of MLEC WT and *Sdc4‐/‐* motility (A) Representative images of migration assays comparing MLECs from WT and *Sdc4‐/‐* mice treated with mesoglycan (0.3 mg·mL^−1^), VEGF‐A (10 ng·mL^−1^), mesoglycan (0.3 mg·mL^−1^) and VEGF‐A (10 ng·mL^−1^) co‐administered. Scale bar = 150 μm. (B) Quantification of EC migration. Magnification 10×. (C) Representative images of tube formation by MLECs from WT and *Sdc4‐/‐* seeded for 12 h on matrigel and MLEC medium 1 : 1 treated with mesoglycan (0.3 mg·mL^−1^), VEGF‐A (10 ng·mL^−1^), mesoglycan (0.3 mg·mL^−1^) and VEGF‐A (10 ng·mL^−1^) together. Scale bar = 100 μm. Analysis of (D) tube length and (E) number of branches calculated by imagej (Angiogenesis Analyzer tool) software. The data represent a mean of three independent experiments ± SEM. *P < 0.05 for all treatments vs. untreated cells. ^#^ *P* < 0.05 for all treatments vs. mesoglycan. ^@^ *P* < 0.05 for all treatments vs. VEGF‐A. ^*P* < 0.05 for *Sdc4‐/‐* treatment vs. WT treatment.

In contrast, the absence of SDC4 leads to reduced EC migration in response to all treatments (Fig. 3A,B), suggesting a role for this proteoglycan in both VEGF‐A and mesoglycan‐driven responses. These results were mirrored when tubule formation in response to matrigel was assayed. WT MLECs showed enhanced response to VEGF‐A and mesoglycan, and this was enhanced when the two were combined. In all instances SDC4 showed a lack response (Fig. 3C–E).

These results suggest that syndecan‐4 may have a role in the pro‐angiogenic pathways stimulated by VEGF‐A and mesoglycan.

### Syndecan‐4 has a role in mesoglycan‐VEGF‐A‐VEGFR2 pathway

**Fig. 4:**
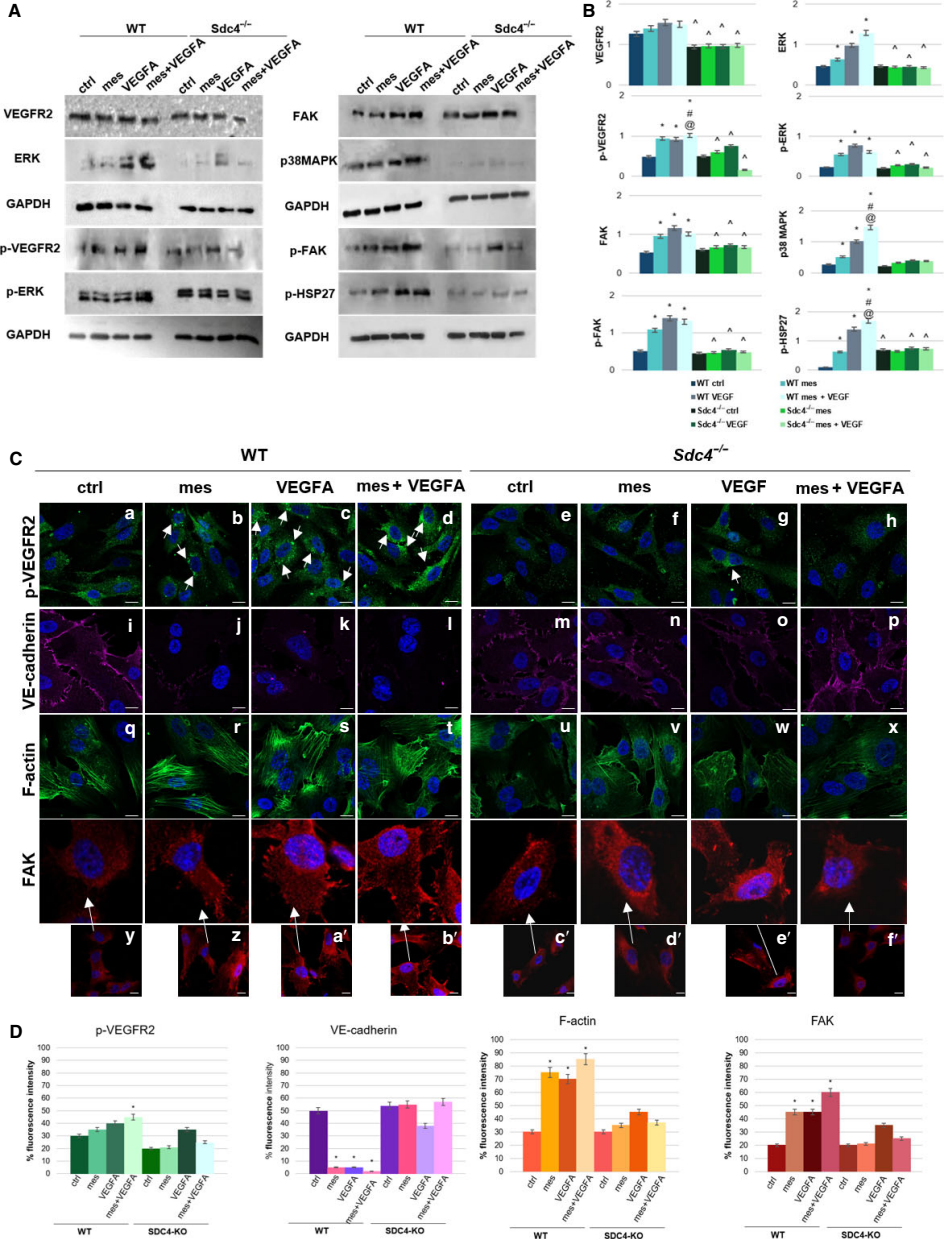
Effect of the absence of SDC4 in VEGFR2 pathway (A) Western blot and (B) quantization of protein extracts from MLEC WT and Sdc4‐/‐ treated or not for 24 h with mesoglycan (0.3 mg·mL^−1^), VEGF‐A (10 ng·mL^−1^) and mesoglycan (0.3 mg·mL^−1^) and VEGF‐A (10 ng·mL^−1^) co‐administered. *P < 0.05 for all treatments vs. untreated cells. ^#^ *P* < 005 for all treatments vs. mesoglycan. ^@^ *P* < 0.05 for all treatments vs. VEGF‐A. ^*P* < 0.05 for Sdc4‐/‐ treatment vs. WT treatment. (C) Immunofluorescence analysis of MLEC WT and *Sdc4‐/‐* in presence or not of mesoglycan (0.3 mg·mL^−1^), VEGF‐A (10 ng·mL^−1^) and mesoglycan (0.3 mg·mL^−1^) and VEGF‐A (10 ng·mL^−1^) together. The cells were fixed and labelled with antibody against p‐VEGFR2 (panels a–h), VE‐cadherin (panels i–p), FAK (panels y–f’, and relative 4× zoom) and with phalloidin (panels q–x). Nuclei were stained with Hoechst 33342 1 : 1000 for 30 min at room temperature (RT) in the dark. Magnification 63 × 1.4 NA. Scale bar = 50 μm. (D) Fluorescence intensity for p‐VEGFR2, VE‐cadherin, F‐actin and FAK signals on MLEC WT and Sdc4‐/‐ cells using ImageJ software. The measurements are determined on ten field images from a single coverslip and randomly selected for three coverslips. *P < 0.05, vs. untreated control.

Having confirmed the pro‐angiogenic effects that mesoglycan‐VEGF‐A exert on WT and not on *Sdc4‐/‐* MLECs, we sort to determine whether genetic ablation of SDC4 had any impact on VEGF‐A/VEGFR2 signalling. WT and *Sdc4‐/‐* MLECs had equivalent levels of VEGFR2 regardless of the treatments. Additionally, measurement of Tyr951 phosphorylation on VEGFR2 in WT MLECs broadly reflected the situation observed on HUVECs in that VEGA treatment alone and in combination elicited more VEGFR2 phosphorylation. Levels of VEGFR2 phosphorylation were at a lower level in *Sdc4‐/‐* MLECs; however, VEGF‐A treatment did elicit a phosphorylation response. Of note combined treatment of *Sdc4‐/‐* MLECs with mesoglycan and VEGF‐A lead to a reduction in VEGFR2 phosphorylation. VEGF‐A alone or with mesoglycan administered to WT MLECs stimulated an increase in ERK and p‐ERK levels. In *Sdc4‐/‐* MLECs, ERK levels of expression remained similar to the untreated control, while its phosphorylated form increased only in the presence of VEGF‐A alone. In addition, increases in p38MAPK and p‐HSP27 were not evident in *Sdc4‐/‐* cells, while in WT cells their phosphorylation increased following VEGF‐A and mesoglycan‐VEGF‐A treatment in concert. FAK and p‐FAK in WT MLECs appeared upregulate particularly after VEGF‐A and mesoglycan‐VEGF treatment, on the contrary, MLEC *Sdc4‐/* did not show a significant alteration, except for VEGF (Fig. 4A,B). The optical density of all the protein bands detected by western blot in Fig. 4A is reported in Fig. 4B.

We next observed via confocal microscopy the localization of proteins involved in EndMT. For p‐VEGFR2, WT cells presented the same trend previously observed in HUVECs. However, as shown in Fig. 4D, in *Sdc4‐/‐* cells the fluorescence intensity of p‐VEGFR2 was lower than in WT cells (Fig. 4C panels a–h) and its expression level increased only in the presence of VEGF‐A (Fig. 4C panel g). VE‐cadherin was clearly visible at the leading edge of WT MLECs only in the untreated control (Fig. 4C panel i) and disappeared with all treatments (Fig. 4C panels j–l). On the contrary, in *Sdc4‐/‐* MLECs, we found a slight decrease of this protein only in the presence of VEGF‐A (Fig. 4C panels m–p). The quantification of the fluorescence intensity of VE‐cadherin is shown in Fig. 4D. Simultaneously, F‐actin was not completely assembled in *Sdc4‐/‐* cells (Fig. 4C panels u–x) as opposed to WT ones. Furthermore, in the latter, actin filaments appeared well organized with mesoglycan, VEGF‐A, and mesoglycan‐VEGF‐A treatments (Fig. 4C panels r–t). Comparing FAK expression between WT and *Sdc4‐/‐* EC, the reduction of focal adhesions was clearly visible in the absence of the proteoglycan (Fig. 4D). FAK appeared as clusters at the boundary of WT MLECs treated with mesoglycan and VEGF‐A (Fig. 4C panels z–a’), but particularly when combined (Fig. 4C panel b’). In contrast, its expression in *Sdc4‐/‐* cells was similar to the untreated control of WT cells (Fig. 4C panel c’), except for the treatments with VEGF‐A (Fig. 4C panel e’).

Taken together, these results showed that mesoglycan act as a bridge among SDC4 and VEGF‐A‐VEGFR2 pathway.

### Annexin A1 is the link between Syndecan‐4 and VEGFR2

In order to better understand the connection, promoted by mesoglycan, between SDC4 and VEGFR2 in enhancing angiogenesis *in vitro*, we investigated the protein AnnexinA1 (ANXA1) as a key regulator of angiogenesis both in pathological and physiological environment [12, 15, 20].

**Fig. 5:**
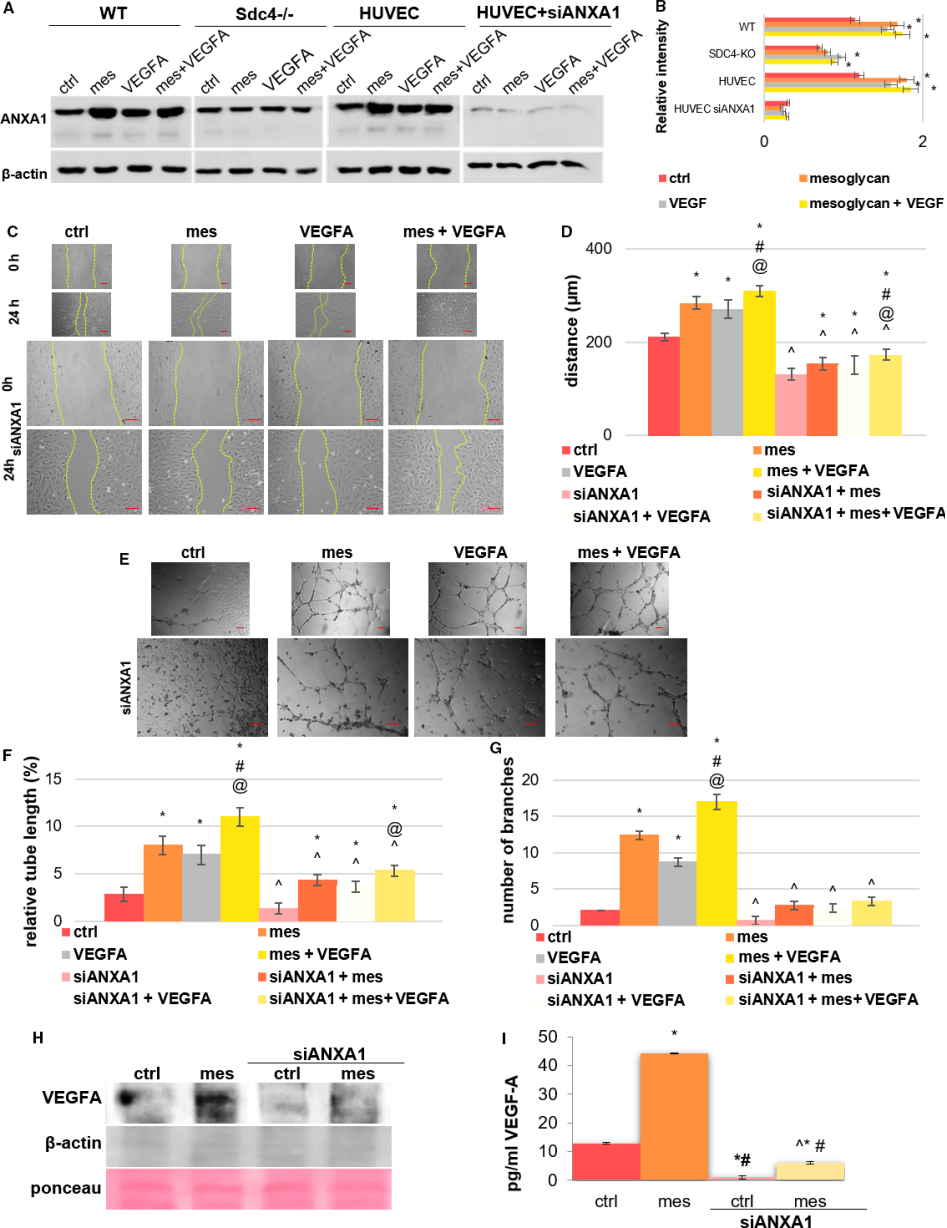
Evaluation of the ANXA1‐mediated effects on mesoglycan‐VEGF‐A treatment on HUVEC motility (A) Western blot and (B) analysis for ANXA1 of total protein extract from HUVEC cells treated or not with mesoglycan (0.3 mg·mL^−1^), VEGF‐A (10 ng·mL^−1^), mesoglycan (0.3 mg·mL^−1^) and VEGF‐A (10 ng·mL^−1^) together, HUVEC treated or not with siANXA1 (100 nm; 48 h) and/or mesoglycan (0.3 mg·mL^−1^; 24 h), VEGF‐A (10 ng·mL^−1^; 24 h), mesoglycan (0.3 mg·mL^−1^; 24 h) and VEGF‐A (10 ng·mL^−1^; 24 h) co‐administered. The shown blots normalized using β‐actin are representative of *n* = 3 experiments with similar results. *P < 0.05 for all treatments vs. untreated cells. ^#^ *P* < 0.05 for all treatments vs. mesoglycan. ^@^ *P* < 0.05 for all treatments vs. VEGF‐A treatment (C) Representative bright field images captured of HUVEC cells (Scale bar = 150 μm) and (D) results from the Scratch Wound Healing Assay at 0 and 24 h from produced wounds treated or not with mesoglycan (0.3 mg·mL^−1^; 24 h), VEGF‐A (10 ng·mL^−1^; 24 h), mesoglycan (0.3 mg·mL^−1^; 24 h) and VEGF‐A (10 ng·mL^−1^; 24 h) together and/or with siANXA1(100 nm; 48 h). Magnification 10×. (E) Representative images of tube formation by HUVEC seeded for 12 h on matrigel and EBM2 medium 1 : 1 and in presence or not of mesoglycan (0.3 mg·mL^−1^; 24 h), VEGF‐A (10 ng·mL^−1^; 24 h), mesoglycan (0.3 mg·mL^−1^; 24 h) and VEGF‐A (10 ng·mL^−1^; 24 h) together and/or with siANXA1 (100 nm; 48 h). Scale bar = 100 μm. Analysis of (F) tube length and (G) number of branches calculated by imagej (Angiogenesis Analyzer tool) software. The data represent a mean of three independent experiments ± SEM. *P < 0.05 for all treatments vs. untreated cells. ^#^ *P* < 0.05 for all treatments vs. mesoglycan. ^@^ *P* < 0.05 for all treatments vs. VEGF‐A. ^*P* < 0.05 for all siANXA1 treatments vs. respective controls. (H) Western blot for VEGF‐A of supernatant from HUVEC cells treated or not with mesoglycan (0.3 mg·mL^−1^) and/or siANXA1. Protein normalization and the check of the sample quality were performed on β‐actin and ponceau levels. The same experimental points have been analysed by ELISA test (I) evaluating supernatants of HUVEC cells. The data represent a mean of three independent experiments ± SEM. *P < 0.05 for all treatments vs. untreated cells. ^#^ *P* < 0.05 for all treatments vs. mesoglycan. ^*P* < 0.05 for all siANXA1 treatments vs. respective controls.

Here, we found an increase of this protein in WT MLECs and HUVECs treated with mesoglycan, compared to *Sdc4‐/‐* cells that presented a small significant rise of ANXA1 only in VEGF‐A treatment (Fig. 5A,B). Based on this, our strategy was to treat HUVECs with small interfering RNA (siRNA) against ANXA1 (siANXA1), by which we obtained a significant decrease in ANXA1, as reported in Fig. 5A,B. We next performed functional experiments on HUVECs using siANXA1 in the presence or absence of mesoglycan, VEGF‐A and in combination. Interestingly, migration and micro‐capillary formation *in vitro* were reduced when ANXA1 had been knocked down in HUVECs (Fig. 5C–G).

From the recent literature [9], we know that the ANXA1‐FPRs binding stimulates the externalization of VEGF. For this reason, we hypothesized that the increase of ANXA1 in HUVEC cells treated with mesoglycan was able to promote the subsequent externalization of VEGF‐A and facilitate the motility. We have revealed via western blot the presence of VEGF‐A in HUVECs supernatant, particularly when treated with mesoglycan (Fig. 5H). To validate the purity of the analysed supernatants, and the absence of cells, we used β‐actin as a technical control; instead, ponceau stain was used for normalization. The different levels of VEGF‐A in HUVEC supernatants have been further revealed by the ELISA (enzyme‐linked immunosorbent assay). Thus, the histogram in Fig. 5I confirmed the high degree of externalization of VEGF‐A in the presence of mesoglycan whose action was notably reduced when ANXA1 expression was decreased by siRNA.

Together, these results provide important insights into the role of ANXA1 as a mediator of angiogenesis promoted by the combination of mesoglycan and VEGF‐A.

### Annexin A1 is released from extracellular vesicles in HUVEC cells treated with mesoglycan

**Fig. 6:**
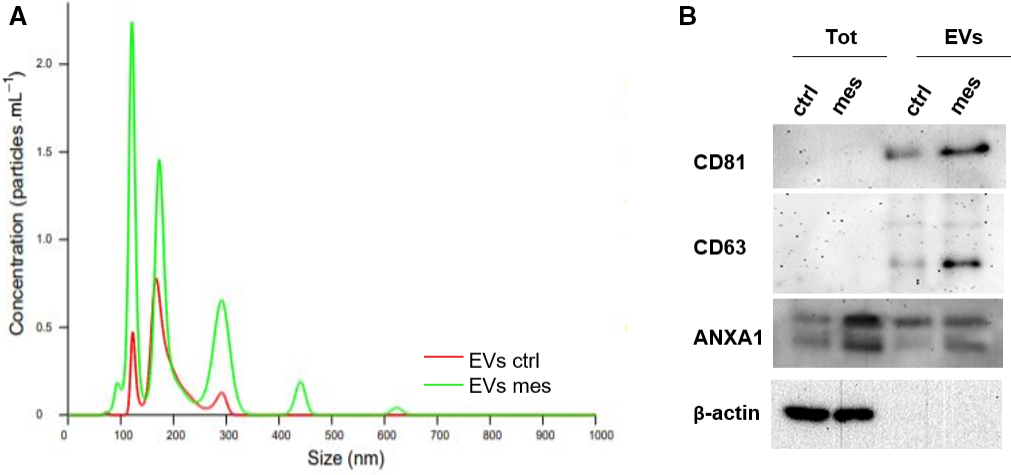
Evaluation of EVs released from HUVEC cells (A) comparison of EVs released from HUVEC cells treated (green line) or not (red line) with mesoglycan (0.3 mg·mL^−1^) by Nanoparticle tracking analysis. (B) Western blot analysis of total protein extracts from HUVEC cells treated or not for 24 h with mesoglycan (0.3 mg·mL^−1^) and from EVs released by the same HUVECs. Cropped blots from full‐length gels are representative of *n* = 3 independent experiments with similar results using antibodies against CD81, CD63, ANXA1 and normalized with β‐actin. The blots were exposed to Las4000 (GE Healthcare Life Sciences).

In our previous work, we have shown that ANXA1 participates in extracellular vesicle (EVs) biogenesis and forms part of their cargo [12, 13]. We started out to observe whether mesoglycan stimulated the release of EVs containing ANXA1 from HUVECs. EVs isolated from HUVECs treated with mesoglycan (EVs mesoglycan) and from the same untreated cells (EVs ctrl) were purified through a serial centrifugation, and their quantity and size were measured using nanoparticle tracking analysis. EVs mesoglycan (green line in Fig. 6A) showed a significant increase in number compared with EVs ctrl (red line in Fig. 6A). The range of the vesicles was from ˜ 50 nm to 630 nm, with a majority of vesicles in the range of 80–150 nm for both the groups (Fig. 6A). This enrichment of EVs between 80 and 150 nm corresponded with the subclass of nano‐vesicles classed as exosomes. To confirm these data, we tested the two groups of vesicles by western blot using CD63 and CD81 as specific exosomal markers [21]. As reported in Fig. 6 B, the expression of CD81 and CD63 was evident only in EV samples and not in HUVECs total lysates. Moreover, the amount of CD81 and CD63 was higher in EVs released in response to mesoglycan, supporting the previous analysis obtained via nanoparticle tracking analysis. We also observed the significant externalization of ANXA1 through EVs mesoglycan, particularly as cleaved form, compared with EVs ctrl (Fig. 6B).

Therefore, mesoglycan promotes a consistent externalization of ANXA1 through EVs.

### Annexin A1 contained in EVs interact with formyl peptide receptors in an autocrine manner

**Fig. 7:**
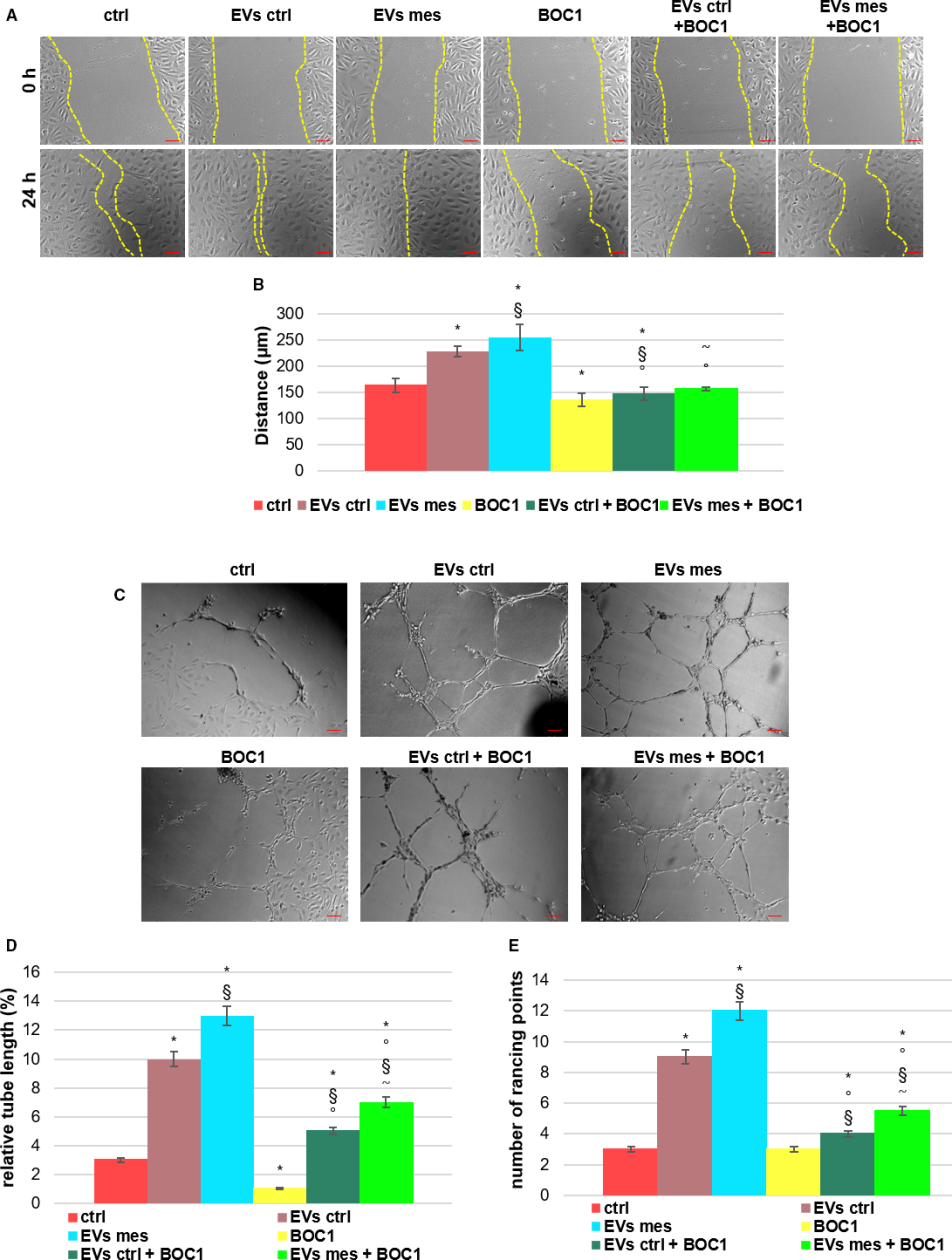
EVs interact via ANXA1/FPRs in autocrine manner (A) Bright‐field images (scale bar = 150 μm) and (B) histogram of wound healing assay of HUVEC cells treated or not with EVs ctrl (1 × 10^6^), EVs mesoglycan (1 × 10^6^), BOC1 (100 μm) and/or their association. (C) Representative images of analysed fields of tube formation assay by HUVEC seeded for 12 h on matrigel and EBM2 medium 1 : 1 and in presence or not of EVs ctrl (1 × 10^6^), EVs mesoglycan (1 × 10^6^) and/or BOC1 (100 μm). Magnification 20×. Scale bar = 150 μm. Analysis of (D) tube length and (E) number of branches calculated by imagej (Angiogenesis Analyzer tool) software. The data represent a mean of three independent experiments SEM. *P < 0.05 for all treatments vs. untreated cells. ^§^ *P* < 0.05 for all treatment vs. EVs ctrl. ^˜^ *P* < 0.05 for all treatments vs. EVs mesoglycan. °*P* < 0.05 for all treatment vs. BOC1.

In [13], we highlighted the autocrine loop ANXA1/EVs/FPRs induced by mesoglycan in keratinocytes. Based on this, we investigated the hypothetic role of the same loop in HUVECs in promoting angiogenesis. HUVEC migration was significantly enhanced with mesoglycan elicited EVs compared with the untreated control (Fig. 7A,B). By utilizing BOC1 at a concentration of 100 µm to block both FPR1 and FPR2 (respectively receptors for and N‐terminal mimetic peptide [22] and ANXA1 [23]), we studied the role of ANXA1 containing EVs. Surprisingly, in the presence of the pan‐antagonist BOC1 HUVEC cell motility was reduced.

We next evaluated the effects of ANXA1 in EVs promoting angiogenesis performing *in vitro* tests of capillary structures. As already seen for migration assay, also in this second process the ability of EVs to stimulate the formation of vessels was confirmed. Notably, EVs mesoglycan promoted a significant number of branching points and the relative tube length compared to EVs ctrl and untreated control. On the contrary, by blocking FPRs these effects were reverted, although the same trend of the experimental points without BOC1 was maintained (Fig. 7C–E).

The results in this section suggested that mesoglycan‐induced ANXA1/EVs/FPRs loop promotes angiogenesis in HUVECs.

### EVs containing ANXA1 promote angiogenesis in a FPRs‐dependent manner

**Fig. 8:**
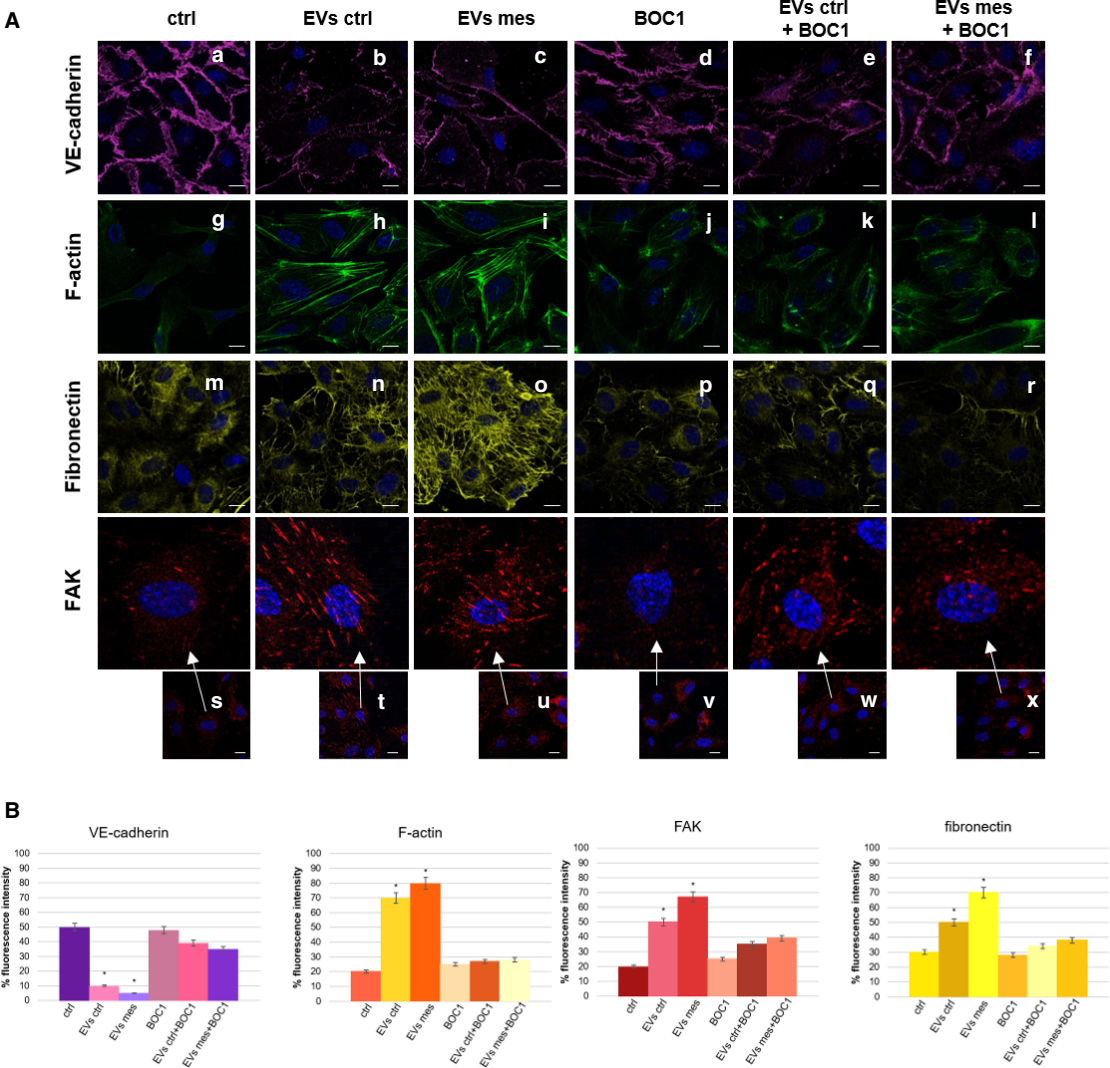
Analysis of EndMT markers on HUVEC cells treated or not with EVs and BOC1. Immunofluorescence analysis of endothelial cells in presence or not of EVs ctrl (1 × 10^6^), EVs mesoglycan (1 × 10^6^), BOC1 (100 μm) and vesicles and BOC1 (100 μm) together. The cells were fixed and labelled with antibody against VE‐cadherin (panels a–f), fibronectin (panels m–r), FAK (panels s–x, and relative ×4 zoom) and with phalloidin (panels g–l). Nuclei were stained with Hoechst 33342 1 : 1000 for 30 min at room temperature (RT) in the dark. Magnification 63 × 1.4 NA. Scale bar = 50 μm. (B) Fluorescence intensity for p‐VEGFR2, VE‐cadherin, F‐actin, FAK and fibronectin signals on HUVEC cells using ImageJ software. The measurements are determined on ten field images from a single coverslip and randomly selected for three coverslips. *P < 0.05, vs. untreated control.

After evaluating that EVs promoted functional effects on motility in autocrine ANXA1‐FPRs‐dependent manner, we investigated EndMT process in the absence of ANXA1 by confocal microscopy. First, as schematized in the histograms in Fig. 8B, we found that VE‐cadherin expression was significantly reduced in HUVECs treated with EVs ctrl and EVs mesoglycan compared to the untreated control (Fig. 8A panels a–c). On the contrary, in the presence of BOC1, this adhesion molecule was visible in the cell junctions, even in presence of the two groups of vesicles (Fig. 8A panels d–f). Based on the variation of HUVECs migration speed, we observed well organized stress fibres in cells treated with mesoglycan EVs (Fig. 8A panels g–i). This cytoskeletal reorganization was not observed when FPRs were blocked (Fig. 8A panels j–l). Moreover, the considerable presence of FAK clusters was influenced by the treatment with EVs. This phenomenon was greatly reduced following the addition of BOC1 (Fig. 8A panels s–x and Fig. 8B). The mesenchymal phenotype acquired by HUVECs is generally characterized by the secretion of ECM proteins such as fibronectin, which supports the elongation of the vessels *in vitro* [24]. We found a strong and structured expression of fibronectin in presence of both types of EVs (Fig. 8A panels m–o). On the contrary, fluorescence intensity levels were significantly reduced when FPRs were blocked in HUVEC cells *in vitro* (Fig. 8A panels p–r). The quantification of the fluorescence intensity of the fibronectin shown in Fig. 8A is reported in Fig. 8B.

Finally, EVs mesoglycan is able to enhance the angiogenesis on HUVECs promoting EndMT.

## Discussion

Numerous studies have explored the use of mesoglycan for the treatment of vascular disease and its efficacious antithrombotic and fibrinolytic effects [15, 25, 26, 28]. We have established the significant *in vitro* impact of mesoglycan in skin wound repair. Mesoglycan is a mixture of GAGs that activate several of the cell types involved in skin regeneration, and these include keratinocytes, fibroblasts, and endothelial cells. Mesoglycan accelerates healing and promotes the formation of granulation tissue, stimulating migration and differentiation of keratinocytes, fibroblast activation and angiogenesis [1, 27].

In order to heal the wounds correctly, the formation of new blood vessels from the pre‐existing vascular system is essential. Endothelial cells, after injury, are stimulated and activated by various pro‐angiogenic factors, including VEGF‐A [5]. In healthy skin, this growth factor is not highly expressed; however, skin injury causes a marked VEGF increase with consequent angiogenesis [28].

In this study, we demonstrate a synergistic effect between mesoglycan and VEGF‐A which enhances the pro‐angiogenic stimuli necessary for wound repair. In HUVECs and aortic ring explants we found that administration of mesoglycan‐VEGF‐A promotes EC migration, tube formation and sprouting compared to a greater extent than the two components individually. The use of mesoglycan and VEGF‐A in combination also resulted significantly more stimulation of VEGFR2‐related signalling pathways that promote angiogenic processes such as cell survival, vascular permeability, proliferation, cytoskeletal rearrangement and cell migration [8]. After the co‐treatment, mesoglycan‐VEGF‐A phosphorylation of several proteins such as p‐ERK, p38‐MAPK and p‐HSP27 is increased. These proteins are important for the actin polymerization [16] and FAK activation necessary for focal adhesion turnover and regulation of migration [29] supporting cell motility.

Our previous studies have highlighted that the mechanism of action of mesoglycan on keratinocytes involves SDC4 [13, 14], a membrane proteoglycan that is also of significant importance in angiogenesis [19].

SDC4 influences the polarization of myoblasts during migration [18], and the reduced motility of SDC4‐deficient cells can affect various cell lines such as fibroblasts [30], hepatic stellate cells [31] and endothelial cells [32]. Endothelial cells from *Sdc4‐/‐* mice exhibited reduced cell migration as compared to WT supporting the hypothesis that it is involved in the pro‐angiogenic effects elicited by the co‐administration of mesoglycan‐VEGF‐A *in vitro*. Obviously, we did not expect the total deprivation of motility in the absence of SDC4, as other pathways can intervene in compensating for the deficiency, one among many the one mediated by VEGFR2. VEGFR2 phosphorylation in response to mesoglycan and VEGF‐A was considerably reduced, and analysis of downstream cellular events such as the redistribution of [33], FAK activation [34] and cytoskeletal remodelling [35] revealed that these were also reduced. Interestingly, mesoglycan alone cannot stimulate any of these pathways.

Prior studies have described the involvement of ANXA1 protein in promoting angiogenesis [11, 12, 15, 20], and we observed an increase of the level of this protein in cells treated with mesoglycan. Hence, to comprehend the role of ANXA1 in our system, we performed functional experiments on HUVEC cell motility by reducing the levels of available ANXA1 using siRNA. Our results showed that with low levels of the protein, endothelial cell motility and tube formation are not promoted, confirming the fundamental role of this protein in angiogenesis.

SDC4 participates to the formation and secretion of EVs [36], and these vesicles may contribute to wound healing [13, 15]. Our previous studies have shown that keratinocytes treated with mesoglycan secreted a large amount of EVs contained ANXA1 in the extracellular environment and promoted angiogenesis *in vitro* [13, 15]. Based on this, we speculated that ANXA1 + EVs could be a link between SDC4 and VEGFR2. Indeed, the treatment of endothelial cells with mesoglycan generated a greater number of ANXA1 + secreted vesicles as was the case with keratinocytes, and we confirmed that this occurred FPRs. We assumed an interaction between ANXA1 and its receptor, just as already demonstrated [13]. This was later confirmed using BOC1 molecule as a pan‐antagonist of FPRs [37]. Indeed, EVs containing ANXA1 can further promote the angiogenic effect of mesoglycan in an autocrine way, but in presence of BOC1, at a concentration of 100 µm namely able to block FPR‐1 and FPR‐2, the effects on this process were not significant. This finding suggests, for the first time, that the interaction between vesicles and endothelial cells occurs through the ANXA1‐FPRs interaction promoting motility and angiogenesis in an autocrine manner, following mesoglycan treatment.

ANXA1‐FPR2 binding can control VEGF‐A secretion in uterine cells [9]. We analysed whether mesoglycan induced secretion of ANXA1 and its interaction with FPRs could promote the externalization of VEGF‐A. Analysing via western blot the supernatant from HUVEC cells treated with mesoglycan, we observed the significant presence of VEGF‐A compared with the untreated cells. Moreover, this growth factor is not secreted in the absence of ANXA1, confirming the needs of this protein to promote the externalization of VEGF‐A.

The involvement of ANXA1 in VEGF‐A release was already demonstrated in another model. Indeed, cardiac macrophages from ANXA1‐KO mice are unable to release VEGF‐A, instead, when these mice treated with ANXA1 showed high amounts of VEGF‐A released from cardiac macrophages [38].

Taken together, our data confirm the positive effects of mesoglycan on angiogenesis stimulation *in vitro* and allowed us to hypothesize a novel mesoglycan‐promoted mechanism. We highlighted the ability of mesoglycan to trigger the activation of three different pathways that convey in HUVEC cells activation. (a) Mesoglycan interacts with the co‐receptors SDC4 and stimulates the expression of EndMT markers, endothelial cell motility and the production of ANXA1‐containing vesicles. (b) These vesicles are then externalized and interact in an autocrine manner with FPRs, amplifying the activation of endothelial cells. (c) The ANXA1‐FPRs interaction results promoting the externalization of VEGF‐A, which in turn stimulates the VEGR2 further supporting angiogenic processes.

How mesoglycan interacts with SDC4 triggering the pro‐angiogenic effects is still unanswered question, so this aspect requires further investigation. A more in‐depth study will be dedicated to the interaction between ANXA1 contained in EVs and FPRs, to understand whether through this bond it allows the vesicles to be internalized by the receptor cells. Moreover, our future perspective is to validate the use of mesoglycan as potential drug triggering pro‐angiogenic effects on blood dermal microvascular cells before translating the research on *in vivo* models.

## Material and methods

### Cell culture and mesoglycan preparation

HUVECs were purchased from American Type Culture Collection (ATCC, Manassas, VA, USA) (ATCC^®^ PCS‐100‐010™) and maintained as reported in [1]. Briefly, cells were maintained at 37 °C in 5% CO_2_−95% air humidified atmosphere and were serially passed at 70–80% confluence. Cells cultured until passage 10.

MLEC from WT and *Sdc4‐/‐* were isolated form wild‐type and syndecan‐4‐null C57BL6 mice (4‐ to 6‐week‐old females) obtained from Charles River Laboratories (Margate, Kent, UK), and kept under pathogen‐free conditions in the Animal Facility of the Queen Mary University of London for 7 days of acclimatization. All experiments were approved in accordance with UK Home Office regulations, under the UK legislation for the protection of animals. Wild‐type and syndecan‐4‐null mice were sacrificed by cervical dislocation and lungs were excised and minced with a scalpel for 5 min. The lung fragments were digested with collagenase (Life Technologies, Carlsbad, CA, USA) for 1 h at 37 °C, then transferred in a petri dish containing 10 mL of MLEC medium (40% Dulbecco's modified Eagle's low glucose medium (DMEM, Life Technologies), 40% Hams F‐12 Medium (Life Technologies), endothelial growth supplement (Sigma‐Aldrich, St. Louis, MO, USA) and 20% of heat inactivated foetal calf serum (Invitrogen, Carlsbad, California, USA). The resulting solution was disaggregated by aspiration through a 19.5‐gauge needle for 4 times. The resulting cell suspension was filtered with a 70‐μm filter and then centrifuged at 300 ***g*** for 5 min. The resultant cell pellet was resuspended in MLEC medium and plated on flasks coated with a mixture of 0.1% gelatin (Sigma Aldrich), 10 mg·mL^−1^ fibronectin (Millipore, Burlington, MA, USA) and 30 μg·mL^−1^ collagen (Advanced Biomatrix, Sea Lion Pl, Carlsbad, CA, USA). After 24 h, the media was refreshed. After 1 week, the endothelial cells were purified by a positive (ICAM‐2; BD Pharmingen, Franklin Lakes, NJ, USA) cell sort using anti‐rat IgG‐conjugated magnetic beads (Dynal, Wiltshire, UK).

Mesoglycan is a natural GAG preparation extracted from porcine intestinal mucosa and is composed of heparan sulfate (47.5%), dermatan sulfate (35.5%), slow‐moving heparin (8.5%) and chondroitin sulfate (8.5%) [27]. Powder of sodium salt mesoglycan was kindly provided by Laboratori Derivati Organici (LDO) S.p.a. (Trino, Italy) and dissolved in the cell medium at an initial concentration of 1 mg·mL^−1^, as previously reported [14, 27]. Mesoglycan administration was established in a dose of 300 μg·mL^−1^ for all performed experiments.

### *In vitro* Wound‐Healing assay

HUVEC and MLEC cells were seeded in a 12‐well plastic plate at 5 × 10^5^ cells per well. After 24 h of incubation, cells reached 100% confluency and a wound was handmade produced in the middle of the monolayer by gently scraping the cells with a sterile plastic p10 pipette tip to create a wound area of about 500 μm [39]. After removing incubation medium and washing with PBS, cell cultures were incubated in the presence of mesoglycan (0.3 mg·mL^−1^, LDO, Laboratori Derivati Organici spa, Trino, Italy), VEGF (10 ng·mL^−1^, VEGF‐165 recombinant human for HUVEC cells and VEGF‐164 recombinant mouse for MLEC, R&D Systems, Minneapolis, MN, USA), BOC1 (100 μM, Bachem AG, Bubendorf, Switzerland) and/or EVs ctrl (1 × 10^6^) and EVs mesoglycan (1 × 10^6^), both isolated from HUVEC. All experimental points were further treated with mitomycin C (10 μg·mL^−1^, Sigma‐Aldrich) to ensure the block of mitosis. The wounded cells were then incubated at 37 °C in a humidified and equilibrated (5% v/v CO_2_) incubation chamber of an Integrated Live Cell Workstation Leica AF‐6000 LX (Leica Microsystems, Wetzlar, Germany). A 10 × phase contrast objective was used to record cell movements with a frequency of acquisition of 10 min on at least 10 different positions for each experimental condition. The migration rate of an individual cell was determined by measuring the distances covered from the initial time to the selected time‐points. Particularly, the analysis was performed on at least 10 different cells randomly selected on each wound edge evaluating the movement highlighted by the sequence of the images taken by the microscope during the experimental time. The distance travelled by the single cell under examination between one side of the scratch and the other can be measured using Leica ASF software (bar of distance tool, Leica ASF software, version Lite 2.3.5, Leica microsystem CMS Gmvh).

### Tube formation assay

A 24‐well plate was coated with Matrigel (Becton Dickinson Labware, Franklin Lakes, NJ, USA) mixed to EGM‐2 1 : 1 for HUVEC cells and MLEC medium 1 : 1 for MLEC WT and *Sdc4‐/‐* on ice and incubated at 37 °C for 30 min to allow gelation to occur. The cells were seeded to the top of the gel at a density of 2 × 10^4^ cells/well in presence or not of the treatments. Cells were incubated at 37 °C with 5% CO_2_. After 12 h, pictures were captured using EVOS^®^ light microscope (10×) (Life technologies Corporation). The length of each tube was measured, and the number of branches was calculated using imagej (NIH, Bethesda, MD, USA) (Angiogenesis Analyzer for imagej) software.

### Western blot

Protein expression was examined by SDS/PAGE, as described in [40]. Briefly, total intracellular proteins were extracted from the cells by freeze/thawing in lysis buffer containing protease inhibitors. Protein content was estimated according to Bio‐Rad protein assay (BIO‐RAD). A total of 20 µg of proteins was analysed using the chemiluminescence detection system (Amersham Biosciences, Little Chalfont, UK) after incubation with primary antibodies against VEGFR2 (rabbit polyclonal; 1 : 10 000; Cell Signaling Technology, Danvers, MA, USA), p‐VEGFR2 (rabbit monoclonal; (Tyr951); 1 : 500; Cell Signaling Technology), ERK (rabbit monoclonal; 1 : 1000; Cell Signaling Technology), p‐ERK (rabbit monoclonal; 1 : 1000 (Thr202/Tyr204); Cell Signaling Technology), p38MAPK (rabbit monoclonal; 1 : 1000; Cell Signaling Technology); p‐HSP27 (rabbit monoclonal; S78; 1 : 250; Cell Signaling Technology); FAK (rabbit monoclonal; 1 : 1000; Cell Signaling Technology); p‐FAK (rabbit monoclonal; (Tyr397) 1 : 1000; Cell Signaling Technology); VEGF (rabbit polyclonal; 1 : 100; Santa Cruz Biotechnologies, Dallas, TX, USA); ANXA1 (rabbit polyclonal; 1 : 10 000; Invitrogen); CD81 (mouse monoclonal; 1 : 500; BD Pharmingen); CD63 (mouse monoclonal; 1 : 500; BioLegend, San Diego, CA, USA); GAPDH (rabbit monoclonal; 1 : 1000 Cell Signaling Technology); and β‐actin (mouse monoclonal; 1 : 1000; Santa Cruz Biotechnologies). The blots were exposed to Las4000 (GE Healthcare Life Sciences), and the relative band intensities were determined using imagequant software (GE Healthcare Life Sciences).

### Confocal microscopy

After the specific time of incubation, HUVEC and MLEC WT and SCD4‐KO were fixed in p‐formaldehyde (4% v/v with PBS) for 5 min, permeabilized in Triton X‐100 (0.5% v/v in PBS) for 5 min, and then incubated in goat serum (20% v/v PBS) for 30 min. Then, cells were incubated with antibody anti p‐VEGFR2 (rabbit monoclonal; (Tyr951); 1 : 100; Cell Signaling Technology); VE‐cadherin (rabbit polyclonal; 1 : 100; Cambridge, UK); FAK (rabbit monoclonal; 1 : 1000; Cell Signaling Technology); and fibronectin (mouse monoclonal, 1 : 100; Abcam, Cambridge, UK), overnight at 4 °C. After 2 washing steps, cells were incubated with anti‐rabbit and/or anti‐mouse AlexaFluor (488 and/or 555; 1 : 1000; Molecular Probes) for 2 h at RT. Other cells were incubated with FITC‐conjugated anti‐F‐actin (5 µg·mL^−1^; Phalloidin‐FITC, Sigma‐Aldrich) for 30 min at RT in the dark. To detect nucleus, samples were excited with a 458 nm Ar laser. A 488 nm Ar or a 555 nm He‐Ne laser was used to detect emission signals from target stains. Samples were vertically scanned from the bottom of the coverslip with a total depth of 5 μm and a 63X (1.40 NA) Plan‐Apochromat oil‐immersion objective. Images and scale bars were generated with zeiss zen confocal Software (Carl Zeiss MicroImaging GmbH, Jena, Germany). For immunofluorescence analysis and quantification, final images were generated using adobe photoshop cs4, version 11.0. Quantifications were performed from multichannel images obtained using a 63× objective using imagej, marking either the cell perimeter or the nucleus as the region of interest and calculating integrated densities per area from the appropriate channel. A minimum of 50 cells were analysed for each data set. The obtained mean value was used to compare experimental groups.

### siRNAs and transfection

The knockdown ANXA1 proteins in HUVEC cells were performed using small interfering RNA (siRNAs) targeting human ANXA1 proteins as reported in [14]. All siRNAs were purchased from IDT (Integrated DNA Technologies Inc., Coralville, IA). The duplex sequences to target ANXA1 were as follows: (a) sense 5′‐GCU AUG AUC AGA AGA CUU UAA UAA T‐3′ and antisense 3′‐UUC GAU ACU AGU CUU CUG AAA UUA AUA‐5′; (b) sense 5′‐GUU GUU UUA GCU CUG CUAAAA ACT C‐3′ and antisense 3′‐UCC AAC AAA AUC GAG ACG AUU UUU GAG‐5′; (c) sense 5′‐AAG UAC AGU AAG CAU GAC AUG AAC A‐3′ and antisense 3′‐GGU UCA UGU CAU UCG UAC UGU ACU UGU‐5′. siRNA Oligo‐Scrambled (Santa Cruz Biotechnology) was used as control at the same concentration. HUVEC cells were initially plated in media containing 10% FBS. After 24 h, the cells were washed once with PBS and transfected or not with siRNAs by Lipofectamine 2000 (Thermo Fisher Scientific, Waltham, MA, USA) according to the manufacturer's instructions. The cells were processed for western blot analysis at 48 h after transfection. The administration of mesoglycan and/or VEGF was performed 24 h after the transfection.

### EVs isolation

HUVEC cells (about 2 × 10^6^ cells) were incubated for 24 h in EGM‐2 (DMEM, Euroclone, Milan, Italy) without FBS treated or not with mesoglycan (0.3 mg·mL^−1^). Then, the supernatant was centrifuged at 4400 ***g*** at 4 °C for 15 min to pellet death cells, followed by a second centrifugation at 13 000 ***g*** at 4 °C for 2 min to remove apoptotic bodies. EVs were enriched by centrifuging at 20 000 ***g*** at 4 °C for 30 min, the supernatant was removed, and pellets were re‐suspended in the selected buffers. The buffer we chose for the resuspension was 100 μL PBS for the administration to cells and nanoparticle tracking analysis 30 μL RIPA lysis buffer to perform Bradford assay. All analyses were performed on fresh isolated fractions.

### Nanoparticle tracking analysis for sizing EVs

Approximately 0.5 mL of EVs (between 10^6^ to 10^9^ vesicles) in suspension was loaded onto the NanoSight NS300 with 488 nm scatter laser and high sensitivity camera (Malvern Instruments Ltd., Malvern, UK); five videos of 90 s each were recorded for each sample. Data analysis was performed with NTA2.1 software (NanoSight, Malvern, UK). Software settings for analysis were the following, Detection Threshold: 5–10; Blur: auto; Minimum expected particle size: 20 nm.

### ELISA for VEGF‐A

After treatments with mesoglycan and siRNA against ANXA1, HUVEC supernatants were collected and the secreted VEGF‐A amount was quantified through the Human VEGF‐A ELISA kit, following the manufacturer’s instructions (Invitrogen).

### Statistical analysis

All results are the mean ± SEM (Standard Error of Mean) of at least 3 experiments performed in triplicate. Statistical comparisons between groups were made using one‐way ANOVA comparing two variables and two‐way ANOVA to compare experimental groups. ANOVA test was followed by the Tukey's multiple comparisons test. Differences were considered significant if *P* < 0.05, *P* < 0.01 and *P* < 0.001.

## Conflict of interest

The authors declare no conflict of interest.

## Author contributions

EP performed the experiments; EP and JW supported in the experimental procedure; EP wrote the whole original draft; EP, RB, and NN carried out the formal analysis; AP, JW MP, and AF executed the revision and the editing of manuscript; AP, MP, and JW provided the funding acquisition. All authors have seen and approved the manuscript.

## Data Availability

The data that support the findings of this study are available from the corresponding author upon reasonable request.
